# Reconciling the conservation of the purple swamphen (*Porphyrio porphyrio*) and its damage in Mediterranean rice fields through sustainable non-lethal techniques

**DOI:** 10.7717/peerj.4518

**Published:** 2018-04-24

**Authors:** Rubén Moreno-Opo, Josep Piqué

**Affiliations:** 1 Deputy Directorate on Environment, Ministry of Agriculture, Fisheries, Food and Environment, Madrid, Spain; 2 Evolution and Conservation Biology Research Group, Universidad Complutense de Madrid, Madrid, Spain; 3 TRAGSATEC, Madrid, Spain

**Keywords:** Agri-environment schemes, Crop damage, Ebro Delta, Rice paddy, Human–wildlife conflict

## Abstract

Resolving human–wildlife conflicts requires the assessment and implementation of appropriate technical measures that minimize negative impacts on socio-economic uses, including agriculture, and ensure the adequate protection of biological diversity. Rice paddies are widely distributed in the western Mediterranean region. Because of their high productivity, they can be a good habitat for waterbirds, including the purple swamphen *Porphyrio porphyrio*, particularly in areas where natural wetlands have been removed or reduced. As a result of its population growth, there have been increasing levels of damage caused by this species in rice fields due to stem-cutting and opening of bald patches in rice fields. With the aim of reducing damage, we evaluated the effectiveness of passive and active measures that would limit access to rice fields and deter/scare away purple swamphens in affected areas of the Ebro Delta (NE Spain). We selected the techniques according to the growth phase of rice and the activity of birds in the rice fields (perimeter fences and clearing vegetation around the rice plots during sprouting and growing phases, and falconry at maturation). There were positive results during the sprouting and growing phases thanks to fences and clearing vegetation, reducing the affected area by 37.8% between treatment and control plots. This would mean an economic savings of 18,550 €/year in compensation payments by regional administrations including the investment in implementing and maintaining passive protection measures. Active deterrence through falconry did not reduce the level of damage. The analysis of purple swamphen home range, activity centers (centroids), and the proportion of locations in and outside of rice fields showed no differences before and after dissuasive practices. These results were influenced by multiple concurrent factors including weather, the structural configuration of the rice plots and their location. In summary, we recommend the establishment of protection measures (perimeter fences + clearing vegetation around the rice plots) to reduce the level of damage.

## Introduction

The resolution of human–wildlife conflicts is a common area of study in environmental management and research. It aims to reconcile the conservation of wild species, especially those most threatened, with socioeconomic uses and human security (Woodroffe, Thirgood & Rabinowitz, 2005; Redpath et al., 2013; Ernoul et al., 2014). Agriculture is one of the most important sectors in which wildlife–human interactions occur (Conover, 2002). There are economic, health, and food security impacts, and the level of severity depends on the type of species involved as well as on the geographical scope (Conover, 2002). These conflicts between people and wildlife in agricultural environments have been the subject of numerous studies and management programs at a global level, with large economic investments from international organizations, mainly on the African continent, even in some cases to alleviate famines when animals behave as pests (Van Huis, 2009; Food and Agriculture Organization, 2012).

Rice *Oryza sativa* L. is a crop affected by wildlife species (Lane, Azuma & Higuchi, 1998; Pernollet et al., 2015) owing to the large number of animal species inhabiting these agricultural habitats due to their high productivity levels, their similarity to natural wetlands and the refuge they provide (Elphick, 2000; Sánchez-Guzmán et al., 2007; Toral & Figuerola, 2010). Rice is one of the most important food crops in the world, with a gross annual production of approximately 715 M tons and an average consumption of 55.2 kg per person/year, although this varies geographically (Muthayya et al., 2014). Among the bird species that inhabit rice fields is the purple swamphen *Porphyrio porphyrio.* It is widely distributed in southern and southeastern Asia, Oceania, sub-Saharan Africa, the Middle East and the Mediterranean basin, and is considered a species of “*Least Concern*” regarding its conservation status (Taylor, 2016). The purple swamphen is linked to wetlands and selects dense masses of marsh vegetation (mainly *Phragmites* spp. and *Typha* spp.) in its Mediterranean range (Taylor & Van Perlo, 1998). The expansion of this species in southern Europe has been remarkable in the last 30 years, shifting from a threatened species for most of the 20th century to common since 1990 (Sánchez-Lafuente et al., 2001), with large groups recorded in several Iberian wetlands in recent years (Bertolero et al., 2016). However, there are not precise population estimates of this species due to the cryptic behavior of its individuals (Pearlstine & Ortiz, 2009).

There are wide areas of rice fields close to natural wetlands which have also been occupied by the purple swamphen in recent times, such as the Guadalquivir marshes, Valencia´s Albufera, the Ebro Delta and the Rhone Delta in Europe, or the Senegal, Nile, and Niger river deltas in Africa (Fasola & Ruiz, 1996; Sánchez-Guzmán et al., 2007; Ibáñez et al., 2010; Toral & Figuerola, 2010; Bertolero et al., 2016). These rice fields are used by the purple swamphen both for feeding on buds (Taylor & Van Perlo, 1998; Taylor, 2016) as well as for shelter and reproduction due to the high coverage generated by the rice plant in developed growth stages (Elphick et al., 2010; Pierluissi, 2010).

The purple swamphen has been rarely studied with respect to the damage it causes to agriculture, unlike other groups such as ducks, flamingos, and some passerines (Tourenq et al., 2003; Van Huis, 2009; Pernollet et al., 2015). This is an emerging issue in the western Mediterranean and, to improve our knowledge about how this damage occurs, it is necessary to take into consideration several behavioral traits of the species. The purple swamphen eats rice plants by cutting stems with its strong beak and manipulating the food skillfully with their digits (Pellis, 2011). The rice plants are damaged by cutting, since the tenderest sprouts are often selected. The plant is not completely cut down, triggering the regeneration of the plant but temporally mismatched with respect to unaffected plants. The purple swamphen also seeks refuge in the vegetation, hiding from potential predators, even at long distances (Blumstein, 2006). It is therefore difficult to apply dissuasive measures during the crop phase when the species feels most protected within the rice field.

The cutting and ingestion of rice seedlings and the opening of bald patches (paths, open areas, nests, etc.) in the rice fields by the purple swamphen are considered by rice farmers as damage resulting in lost income. Thus, compensatory payments for farmers at different Spanish wetlands are being made by the regional administrations under the existing regulations (Jefatura del Estado, 2007). For example in the Ebro Delta, administrators had paid up to 203,450 € per year from 2003 to 2016 to compensate for damages on about 21,000 ha of rice fields. As the purple swamphen is specially protected by national and European legislation, actions that may affect its favorable conservation status, such as lethal control or captures and translocations, cannot be undertaken without first showing the absence of any satisfactory alternative measure to avoid or alleviate crop damage.

In the present study, we aimed to evaluate the effectiveness of three measures of crop protection (vegetation management, physical barrier, and falconry) to prevent and mitigate damage caused by the purple swamphen. These measures have not been scientifically tested to date, given the novelty of the conflict in the Mediterranean area. In addition, we also determined the factors influencing the impact of the purple swamphen on rice fields associated with natural wetlands in the Mediterranean region, quantified damage to rice fields as a function of the growth phase of the crop, and determined the land use of GPS-tracked purple swamphens within and outside rice fields associated to deterrence activities with falconry. The overall purpose of this work is to provide information on alternatives aimed at reconciling the presence of this wild species with rice cultivation, minimizing damage and establishing management proposals in sensitive areas to maintain both economic benefits from agronomic yield and biodiversity levels.

## Methods

### Study area

**Figure 1:**
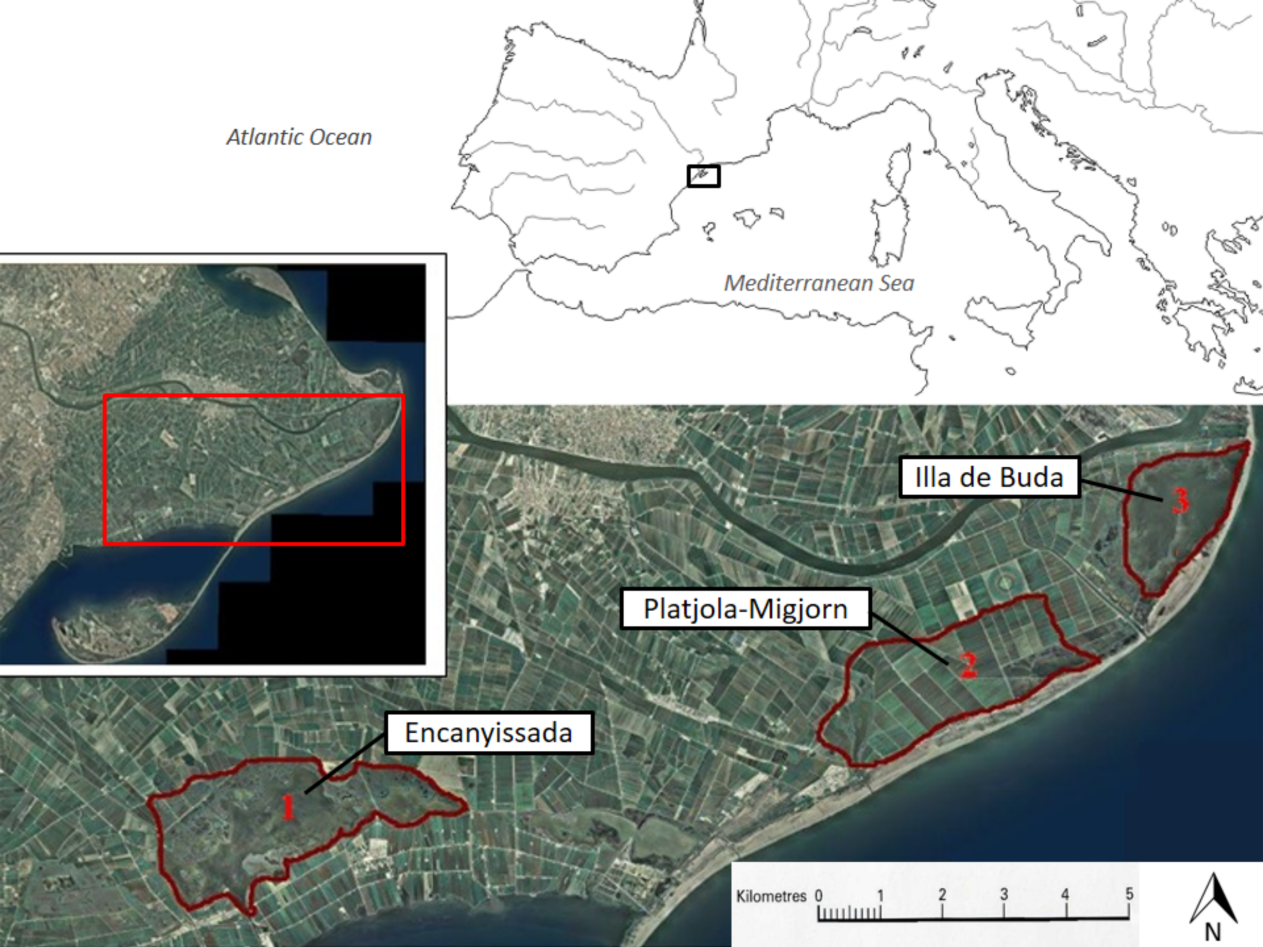
Study area. Areas selected to assess the effectiveness of measures to mitigate damage by the purple swamphen *P. porphyrio* on rice crops in the Ebro Delta (NE Spain). The selected areas include rice field plots and are identified as: 1, Encanyissada; 2, Platjola-Migjorn; and 3, Illa de Buda.

The study was carried out in areas of the Ebro Delta (Catalonia, NE Spain, Fig. 1) most severely affected by purple swamphen damage. It is one of the most important coastal wetlands in the western Mediterranean, with an area of 320 km^2^ containing habitats rich in biological diversity including both natural (rivers, sea, bays, beaches, dunes, riparian forests, coastal lagoons, river marshes) and anthropic (mainly salt pans and paddy fields) areas (Ibáñez et al., 2010). As a result, the Ebro Delta was declared a Natural Park and Natura 2000 and was included in the Ramsar list (Ibáñez et al., 2010).

Rice paddies occupy 65% of the Ebro Delta area and, together with tourism, constitute the main economic resource for the approximately 15,000 local residents. In this area, an abundant population of purple swamphen has been recorded (Bertolero et al., 2016), mainly distributed in natural wetlands and their surroundings, including rice fields. For the purpose of this study, we selected specific areas (three in total, called *Encanyissada*, *Platjola-Migjorn*, and *Illa de Buda*; Fig. 1) within the Ebro Delta based on the following criteria: (1) higher number of farmers demanding compensation compared to the whole Ebro Delta, (2) similarity in environmental traits, because all the studied rice paddies on the study area are in contact with natural wetlands, (3) regular presence of purple swamphen, (4) similar rice harvesting patterns (conventional rather than organic farming practices), and (5) presence of hunting activity towards game species of waterfowl (but not the purple swamphen, as it is a strictly protected species). Within the three areas, we selected 22 different rice plots, obtaining a sample of 49 experimental units in the three years of study (Table 1; Table S1). Of these units, 29 were treatment plots (where preventive measures were applied) and 20 were controls (without measures against the purple swamphen; Table 1; Table S1).

**Table 1:** Number of “Treatment” and “Control” plots.

Study area	2013		2014		2015	
	V + PB	C	V + PB + F	C	V + PB + F	C
*Encanyissada*	4	1	2	2		
*Platjola-Migjorn*	4	1	3	3	4	4
*Illa de Buda*	4	1	3	3	5	5
	Treatment	Control	Treatment	Control	Treatment	Control
Total	12	3	8	8	9	9

**Notes:**

Number of “Treatment” plots selected to study the effectiveness of different preventive techniques (V, vegetation management at the edges of the plots; PB, physical barrier techniques using plastic nets as a fence; and F, falconry) that would limit access to rice fields by purple swamphens *P. porphyrio* and plots without preventive techniques (C, “Control”) in the three study areas of the Ebro Delta (NE Spain) during 2013, 2014, and 2015.

### Study design and variables considered

We conducted fieldwork during the rice growing seasons (late April to September) of 2013, 2014 and 2015. The sampling unit was a crop plot (defined as a parcel covered by rice separated from other parcels within the three selected areas). The plots had the following common characteristics: (1) an area between 1 and 5.5 ha, (2) square or rectangular shape, (3) contact of some of their sides with dense masses of natural marsh vegetation, and (4) rice crops of conventional agronomic types. We selected a different number of study plots per year (mean 6.1 ± 1.9; range 4–10) within each of the three areas, dividing the total number of plots into *treatment* plots (where dissuasive method were implemented), and *control* plots (without dissuasive measures). All the plots considered were located at a minimum distance of 50 m from other study plots.

On each plot, we evaluated the following explanatory variables: (1) *plot area*, (2) *length of border adjacent to a reedbed*, and (3) the type of plot: *treatment* vs. *control* (see below). The response variable was the *percentage of rice area damaged* by the purple swamphen with respect to the total area of the plot (see below), taking into account the different stages of rice development, to check the effectiveness of the implemented measures along the crop maturation cycle: rice *sprouting* (seedlings between 5 and 10 cm height); rice *growing* (plant height between 10 and 40 cm), and rice *maturation* (plants taller than 40 cm, until harvest). We defined these stages based on the use of rice fields by the purple swamphen: during the *sprouting* phase, purple swamphens enter the rice fields exclusively to feed, returning to areas with natural vegetation to breed and to shelter themselves against disturbances or potential predators. During the *growing* phase, the purple swamphen comes and goes from the rice fields and damage is caused by the cutting of stems that prevent growth at the same rate as unaffected plants and by the opening of corridors and paths. Finally, from the *maturation* stage to the crop harvest, the purple swamphen remains within the rice fields without moving to natural wetlands and engages in life-cycle activities such as feeding, resting and breeding.

We did not evaluate the relative abundance of purple swamphen on the rice fields due to the lack of standardized census methods applicable to our objectives. Variables that could influence detection (i.e., time activity patterns, shyness, territorial behavior, vocal activity) are not well known, preventing comparisons of density among the different study areas and plots. Therefore, we used a categorical variable (continuous presence in the plot vs. not continuous presence) and removed from our analyses any plots in which purple swamphens were not detected during field visits to avoid biased estimates of relative abundance of the species, even though the abundance of the target species influences the level of damage (Canavelli et al., 2014).

### Test of preventive measures

**Figure 2:**
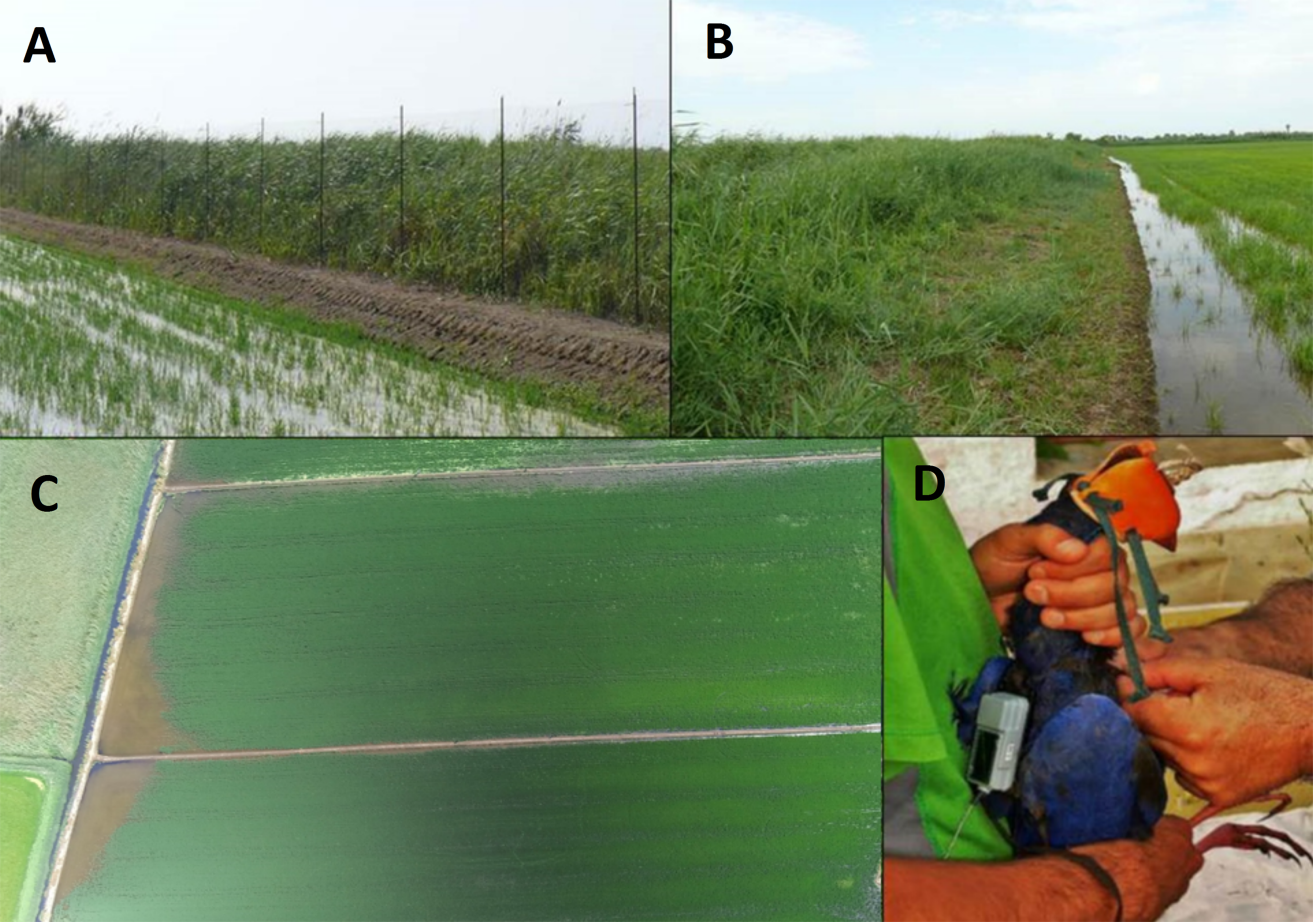
Preventive measures implemented. Tested measures to mitigate damage by the purple swamphen *P. porphyrio* on rice crops in the Ebro Delta (NE Spain): fencing of rice plots on perimeter borders in contact with natural vegetation (A), and clearing of natural vegetation from the borders of rice plots in contact with natural vegetation (B). Aerial photo of rice plots for the delimitation of affected polygons—bald patches in brown color contrasting with green rice plants- due to purple swamphen feeding and trampling (C). GPS-GSM transmitter attached to a purple swamphen to evaluate the effectiveness of falconry as a purple swamphen deterrent in rice fields (D).

We evaluated the efficacy of deterrence measures for each growth stage of rice. During the *sprouting* and *growing* stages (Table 2), we evaluated alternatives for reducing the movement of purple swamphens between feeding (paddy fields) and refuge areas (natural wetlands): (1) *vegetation management at the edges of the plots*, namely periodic clearing and cutting of the natural herbaceous vegetation to avoid intermediate areas of refuge between wetlands and rice fields (Fig. 2) and, (2) *physical barrier* using plastic nets used as a fence to avoid access of swamphens to the rice plots from the areas of refuge. Plastic nets were 2 m high and were installed at the edge between the treatment rice plot and the reedbed, supported by metal poles every 4 m. To avoid entry via the lateral edges of the plot, the mesh was extended by 10–20 m on each side of the plot (Fig. 2). These systems remained in place from the time of sprouting (approximately 20 days after rice sowing) to 10–15 days before harvesting. Biweekly visits were made to ensure their proper functioning and to replace equipment when necessary.

**Table 2:** Type of preventive techniques.

Growth stage of rice	Preventive technique		
	V	PB	F
*Sprouting*	yes	yes	no
*Growing*	yes	yes	no
*Maturation*	no	no	yes

**Notes:**

Type of preventive technique (V, vegetation management at the edges of the plots; PB, physical barrier techniques using plastic nets as a fence; and F, falconry) implemented (yes/no) to limit access to rice fields by purple swamphens *P. porphyrio* during each of the three growth stages of the rice considered (*sprouting*, seedlings between 5 and 10 cm height; *growing,* plant height between 10 and 40 cm; and *maturation*, plants taller than 40 cm, until harvest) in the Ebro Delta (NE Spain).

During the *maturation* phase, when rice paddies are a safe refuge for the birds, we evaluate the efficacy of *falconry* as a method to frighten and deter purple swamphens located within rice fields by means of raptor flights over the treatment plots (Table 2). This technique consisted of a technician walking on the selected plots, carrying a domesticated Harris’ hawk *Parabuteo unicinctus*. These birds, thanks to their previous training, searched for the purple swamphens potentially present along the route on treatment plots from their perches on the falconer or through prospecting flights. Once the purple swamphens were detected, the Harris’ hawk attacked and scared them away. The evaluation was conducted in alternate one-week sessions, starting in the third week of July throughout August for a total of three weeks of evaluation per year in 2014 and 2015. Six consecutive days of deterrence with falconry were carried out per week, except in two weeks in which seven days were performed, with a total of 38 days. Each working day lasted a total of 10 h, equally divided among the different treatment plots and investing approximately 1 h per plot. The schedule was directed to the first 6 h after sunrise and the 4 h before sunset. All the treatment plots were visited daily by the falconer, alternating the time of treatment in each plot between different days. We used six different individual Harris’ hawks suitably trained for this task and alternated during the day to avoid fatigue.

We chose not to study other possible preventive systems, either because of their non-compliance with current legislation (e.g., lethal control) or their lack of applicability due to their economic costs or the required technology. Specifically, we ensured that tested systems would not infringe on the legal protection regime of the purple swamphen at the European level (Birds Directive 2009/147/CE and Bern Convention). Thus, we avoided systems that impacted the survival of purple swamphens (live or dead capture, shooting, changes in natural habitat, etc.). Additionally, we tested techniques, such as decoys of predator species (eagle owl *Bubo bubo* and buzzards *Buteo* sp.), repelling sounds (BirdXpeller and Sentinel models) and LED strobe lights powered with conventional (AA) and/or rechargeable batteries (22A–70A) during the first year of study in the three selected areas. However, they were ultimately discarded for additional study in subsequent years due to the adverse effects of humidity and salinity on the performance of the electronic systems and their high economic costs, which made them unfeasible for use by farmers.

Complementary to the evaluation of the effectiveness of each technique, 10 purple swamphens were captured using falconry in July 2015 at two of the study areas (*Illa de Buda* and an adjacent area to *Platjola-Migjorn* called *Riet Vell*), for the attachment of GPS transmitters. Two Harris’ hawks, with talons filed to reduce injury, capture swamphens within 20 m of field technicians who were able to remove the prey before injuries occurred. None of the 10 purple swamphens captured with this method suffered injuries affecting their survival or subsequent activity. The live capture of the purple swamphens was authorized by the Generalitat de Catalunya government as competent authority (SF/005/2013, Departament de Medi Ambient i Habitatge and Delta de l´Ebre Natural Park). The GPS transmitters (GPS-RF, Solar 28 g, Gruenwald, Germany, www.e-obs.de; Fig. 2) were used to: (1) determine the home range of purple swamphen individuals within the rice fields, (2) evaluate potential shifts in geographic locations, namely the distance between centroids, and the extension of home ranges before and after the disturbances provoked by falconry, and (3) assess the global effectiveness of falconry as a deterrent for purple swamphens. The transmitters were programmed with a frequency of one position every 20 minutes during the daytime (18 h) and one location every hour during the night period (6 h). The data recorded by the GPS transmitters were downloaded with a base station less than 500 m away, once a week in July, August, and September 2015. The extent of the home ranges (kernel polygons 95%; Hemson et al., 2005) and distances between centroids of the different considered periods (Moorcroft, Lewis & Crabtree, 1999) were determined. Purple swamphens were also aged, sexed (Bertolero et al., 2016), measured, banded with plastic leg rings allowing distant reading of their identification codes and then released in the treatment plots in which they had been captured.

### Crop damage estimation

Aerial photographs of the monitored plots in the middle of each of the three periods (*sprouting*, *growing*, and *maturation*) were taken as a technique for evaluating purple swamphen damage to rice fields. A remote control balloon in 2013 and a drone in 2014 and 2015 with an integrated digital photo camera were utilized. Pictures of each plot had a sufficiently high resolution (>12 MPix) for subsequent analysis and were always taken from the same geographical points and at a height of 50 m above the ground (Fig. 2). We obtained a total of 147 aerial photos of rice plots. We georeferenced the photographs according to the real measurements of the plot and analyzed them using a geographic information system (ArcMap10.4, Esri, Redlands, CA, USA). Thus, we calculated the plot area potentially impacted by the purple swamphen through the delimitation of *affected polygons* constituted by bald patches and areas without rice attributable to both trampling and the cutting of stems by the purple swamphen.

### Statistical analyses

We used general linear mixed models to evaluate the relationship between explanatory and response variables. In order to determine which plot traits influenced the damage of purple swamphens to rice, we analyzed the joint influence of the *area, plot identity*, *year* (these latter two variables nested), the total *area* of the plot, the *length of border adjacent to a reedbed*, the *type of plot* applied (*treatment* or *control*) and the *growth phase* (*sprouting*, *growing* and *maturation*) on the *percentage area of rice crop damaged*. To avoid pseudoreplication within the same plots in different years we nested *plot identity* and *year* factors and randomly changed their consideration as treatment or control in the different years of study (Table S1).

Additionally, we performed (1) analyses of variance (one-way ANOVA) to check the differences in home range before and after the implementation of deterrence activities with falconry and between sexes of GPS-tracked purple swamphens (these analyses could not be carried out jointly in a two-way ANOVA due to small sample size), (2) frequency analysis (χ^2^) of levels of deterrence (% of swamphens put to flight) among the different study areas, and (3) paired-sample comparisons (*t*-tests) to assess the differences in the distance of tracked purple swamphens between centroids at the different periods of study (from July to September) and the proportion of locations in and outside of rice fields before and after the deterrence activities with falconry. All analyses were conducted with the software Statistica 7.0 (StatSoft, Tulsa, Oklahoma, USA).

## Results

### Purple swamphen damage in the rice fields

The annual mean ± SD of the area affected by purple swamphen damage in the three analyzed areas was 0.19 ± 0.33 ha per plot at the end of the maturation period (*Encanyissada* 0.32 ± 0.37 ha, min 0.03 ha, max 1.24 ha; *Platjola-Migjorn* 0.25 ± 0.44 ha, min 0.002 ha, max 1.47 ha; and *Illa de Buda* 0.08 ± 0.11 ha, min 0.003 ha, max 0.32 ha) which represented 9.46% ± 16.64% of the area of farmed rice per plot (a total of 137.8 ha of cultivated rice during our study in all considered plots). The percentage area damaged per plot depended on the plot and the year (e.g., during *sprouting* phase 15.7% ± 16.7%, *n* = 15 in 2013; 37.1% ± 29.1%, *n* = 16 in 2014; and 10.3% ± 2.3%, *n* = 18 in 2015; Table 3) and varied among the different studied areas with *Encanyissada* and *Platjola-Migjorn* being the most affected (*F*_2,42_ = 3.136; *p* = 0.051; 6.7% ± 0.11% per plot, *n* = 9 for *Encanyissada* and 25.0% ± 30.7% per plot, *n* = 19 for *Platjola-Migjorn*; Table 3).

**Table 3:** General Linear Mixed Model.

Explanatory variable	Effect	Type of variable	Sum of squares	Degrees of freedom	F	p
*Intercept*	Fixed		183.44	1	1.87	0.1748
Area	Random	Categorical	713.66	2	3.91	0.0234
Plot identity*year	Random	Categorical	9369.61	22	4.67	0.0000
Type of plot	Fixed	Categorical	295.55	1	3.24	0.0750
Growth phase	Fixed	Categorical	332.46	2	1.82	0.1672
Plot area	Fixed	Continuous	190.83	1	2.09	0.1513
Length of border adjacent to a reedbed	Fixed	Continuous	1,223.28	1	13.42	0.0004
*Error*			8291.69	91		

**Notes:**

General linear mixed model (GLMM) to assess the joint effect of different explanatory variables on the *percentage area of rice crop damaged* by the purple swamphens *P. porphyrio* in the Ebro Delta (NE Spain). The statistics results are shown for each studied factor: *area* (Encanyissada, Platjola-Migjorn, Illa de Buda), *plot identity* and *year* (2013, 2014, and 2015), *type of plot* (treatment/control), *growth phase* (sprouting, growing, maturation), *plot area* (in ha) and *length of border adjacent to a reedbed* of a plot (in m).

### Relationship of purple swamphen damage to rice field characteristics

**Figure 3:**
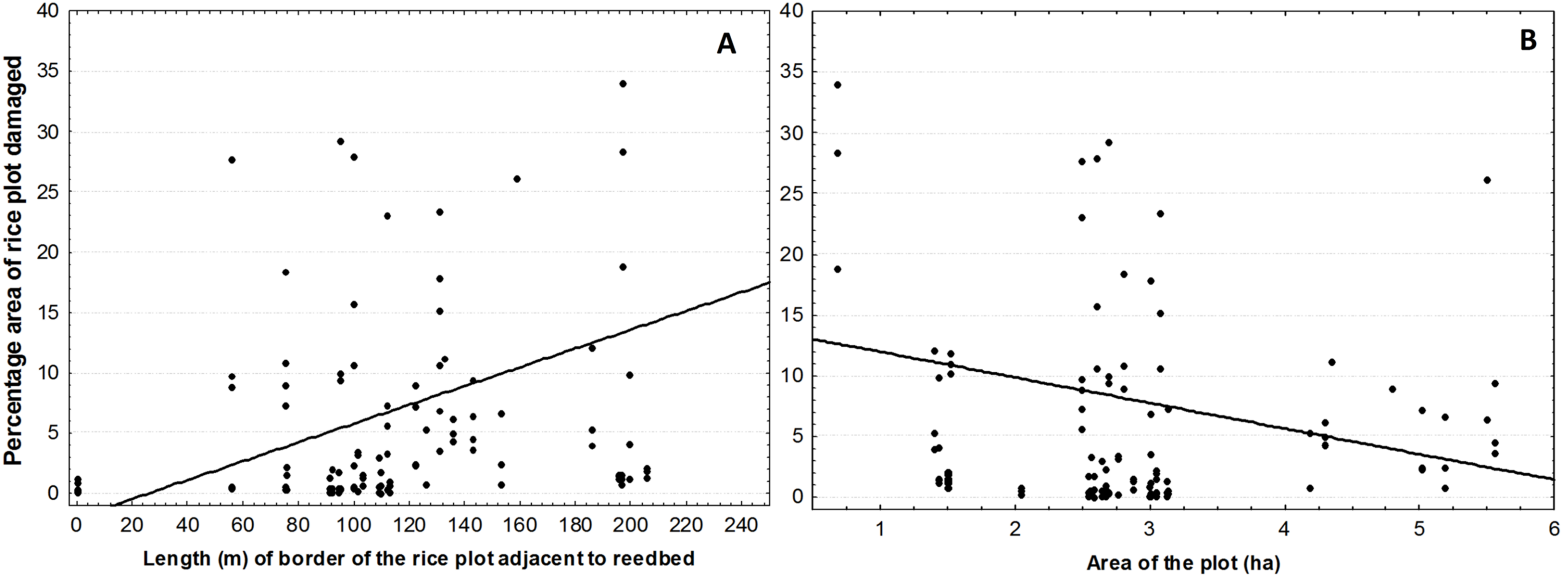
Relationship between the percentage area of damaged rice plot and length of border in contact with reedbed and area of the plot. Relationship between the percentage area of damaged rice plot by the purple swamphen *P. porphyrio* during the three growth phases considered (sprouting, growing, and maturation) and the length in meters of the rice plot borders in contact with natural vegetation, mainly *Phragmites* sp. (A; *r*^2^ = 0.177; *p* < 0.001), as well as the area in hectares of the plot (B; *r*^2^ = 0.027; *p* = 0.067).

The damage caused by the purple swamphen was related to the characteristics of the plot. A longer *length of border adjacent to a reedbed* of the plot was associated with an increased percent damaged area (Table 3; Fig. 3A). The percent damaged area showed a marginally non-significant tendency to decrease with plot size (Table 3; Fig. 3B).

### Relationship of damage to preventive measures

**Figure 4:**
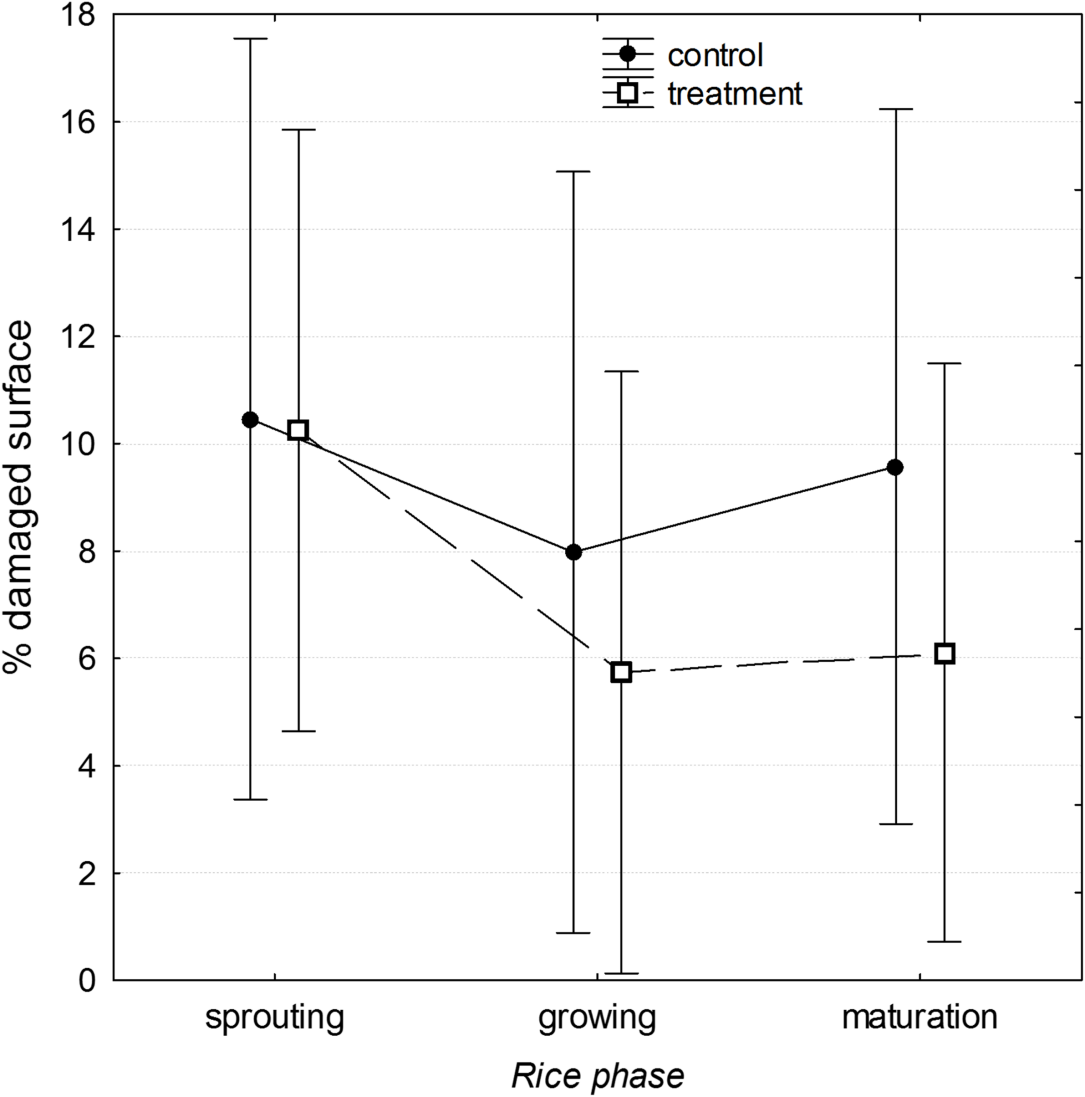
Trend of the mean percentage ± 95% of confidence interval of rice plot area damaged. Trend of the mean percentage ± 95% of confidence interval of rice plot area damaged by the effect of the purple swamphen *P. porphyrio* in the Ebro Delta (NE Spain) between plots with preventive and mitigating measures (*Treatment*) and plots without measures (*Control*), during the different considered phases of rice growth (*sprouting*, *growing,* and *maturation*).

There were marginally significant differences in the percentage of damaged area between plots with and without all tested preventive measures considering all estimates performed (7.3% ± 12.6%, *n* = 87 in plots with preventive measures, and 9.3% ± 15.2%, *n* = 60 in plots without preventive measures; Table 3). Regarding the growth phases, there were no global differences in the damaged area considering all studied plots jointly (Table 3). Nonetheless, between *sprouting* and *growing* periods, the reduction in damage was significantly greater in plots with mitigation measures that included physical barriers and vegetation management (Table 2) compared to control plots (*F*_1,37_ = 4.343; *p* = 0.044; Fig. 4). As a result, treatments reduced the area damaged at the final stage of *maturation* by 37.8% (9.8% ± 14.5%, *n* = 20 in control plots vs. 6.1% ± 12.0%, *n* = 29 in treatment plots, an absolute reduction of 3.7%; Fig. 4).

With respect to falconry which was implemented only during the maturation stage of the rice, in 2014 the 207 flying actions managed to frighten purple swamphens on 32 occasions. There were significant differences in the number of individuals put to flight as a function of the three study areas (χ^2^_2_ = 10.697; *p* = 0.004), which was higher in areas with smaller plots (i.e., *Platjola-Migjorn n* = 21 flights > *Illa de Buda n* = 10 flights > *Encanyissada n* = 1 flight). In 2015, the falconry focused on the 10 purple swamphens that were captured and GPS-equipped. According to the criteria used by Bertolero et al. (2016), all of them were adults (from their second calendar year), including five males, four females and one of unknown sex. After their capture in July, three alternating weeks of falconry directed at the 10 swamphens were carried out until the end of the experiment on September 17th. For all the studied birds from July to September, the home range was 71.9 ± 39.2 ha, and was similar for males and females (69.9 ± 8.7 ha and 74.0 ± 63.4 ha respectively; *F*_1,6_ = 0.016; *p* = 0.902). Between the considered periods (before and after falconry), there were no significant differences in the home range (*F*_1,12_ = 1.106; *p* = 0.313), or when considering the sex of the birds (*F*_1,10_ = 1.627; *p* = 0.230). A distance of 144.1 ± 136.6 m was recorded between the centroids of the locations of the equipped purple swamphens before and after falconry. These values varied slightly and not significantly between the different deterrence periods (July–August 91.6 ± 115.2 m and August–September 128.0 ± 126.0 m, *t*_6_ = −0.384; *p* = 0.714). In this sense, the percentage of locations in and outside of rice fields between pre- and post-deterrence did not differ significantly (*t*_7_ = 1.119; *p* = 0.300). Thus, purple swamphens continued using rice fields frequently (87.5% ± 6.5% of locations) despite the deterrence actions.

## Discussion

According to our results, damage attenuation in rice paddies of the Ebro Delta was decreased in protected plots just before the rice harvest (Fig. 4). But in addition to the studied variables, other factors influenced our results. These are complex and difficult to control in research experiments. Thus, the high variability in the level of damage produced from year to year, the weather conditions, the structural configuration of the rice plots, their location, the type of rice sown or the management and agronomic techniques are traits to be considered. Mitigating wildlife damage in agriculture is challenging and the results obtained in tests on a global scale are frequently far from definitive or satisfactory (Treves, Wallace & White, 2009; Dickman, 2010). Crops are often available to animals over large areas and for long periods, making it difficult to apply sustainable and effective preventive techniques at all spatiotemporal levels (Woodroffe, Thirgood & Rabinowitz, 2005; Canavelli et al., 2014). This is especially true for some damaging vertebrates because of their adaptive capacity to anthropized environments, such as crops (Battin, 2004; Toral & Figuerola, 2010, Bertolero et al., 2016).

We could not consider the relative abundance of the purple swamphen as a factor determining the level of damage in rice paddies because it is inconsistently detectable, both visually and vocally, exhibits an aggregated distribution around favorable areas and shows unknown dynamics of habitat use between seasons. Although we carried out preliminary tests with camera traps to assess the frequency of transit between zones of natural vegetation and rice fields (O’Brien & Kinnaird, 2008) as a comparative index of the level of use of each study plot, we found several limitations preventing the acquisition of useable data: (1) the requirement for simultaneous availability of excessive numbers of cameras due to the length of plot borders in contact with reedbeds, (2) the possibility of aerial access to the rice fields not subject to any known pattern increased the biases in comparisons between plots, and (3) the rapid deterioration of the equipment due to the humidity and salinity.

### Tested preventive measures and damage assessment system

Techniques creating a barrier between the rice crop and the natural environments offered the best results. The plastic nets used as a fence provide a visual and physical barrier to purple swamphens because they usually move on foot (Taylor & Van Perlo, 1998; Treves, Wallace & White, 2009). Fences should be maintained in good condition to prevent the opening of gaps in the net, especially close to the ground, which could be used for access by the purple swamphen and other species (e.g., common moorhen *Gallinula chloropus*). The establishment of large areas without natural vegetation around rice plots enhances these barriers, as purple swamphen are unlikely to cross open areas due to their cautious nature (Blumstein, 2006). The effectiveness of this measure is temporary and has positive effects during the *growing* phase until the rice is about 40 cm high. After this phase, during *maturation*, the purple swamphen no longer leaves the rice field, remaining inside, so that neither fencing nor the clearing of vegetation will be effective. The marginally significant differences between plots with and without treatment (Table 3) could suggest that barriers during the early phases of rice growth could still produce a reduction in total damage.

The use of falconry was shown to be ineffective in mitigating damage. In spite of the continuous attacks, the purple swamphens did not modify their habitat use, remaining in the same areas in the rice fields. The breeding that occurs in the rice fields from August onwards, settles the birds around invariant areas around the nest (Boyd et al., 2014). Falconry, in addition, requires considerable prospecting effort and specific training for targeting the purple swamphen. Unintentional captures of other birds present in the rice paddies, such as molting ducks, moorhens or different waders, may occur. Nonetheless, this technique was useful for the live capture of the target species for its study in hand and for applying telemetry equipment.

Our results could include biases that would weaken the effect of the applied protection measures, due to reduced accuracy in the delimitation of *affected* vs. *not affected polygons* at the final stages of rice growth, as Fig. 4 indicates. It is necessary to take into consideration that rice plants recover and compensate initial damage throughout the whole process of growing and maturation. The initial bald patches considered as damaged are mostly replaced by resprouted plants so the overall delimitation of *affected* vs. *not affected polygons* decreases jointly at all rice paddies. Nevertheless, this bias should be similar in all study areas so that comparisons between control and treatment plots would be valid. We used high-quality aerial photography at a 50 m in height as the most objective, replicable and quantifiable damage assessment technique (Louhaichi, Borman & Johnson, 2001; Morgan, Gergel & Coops, 2010). This technique allowed us to differentiate between treatment and control plots during the early stages of rice development, since growing areas were markedly greener in comparison to non-growth, damaged areas (usually brown). At more advanced phases, discriminating these differences was more challenging due to the regrowth of initially damaged rice, which results in a green color similar to that of well-developed rice. Therefore, another approach using aerial photography at a lower height and increased resolution is required to identify the degree of maturation of the rice and to identify other natural, non-rice plants (i.e., bulrushes *Scirpus* spp.).

## Conclusion

The preventive measures applied during the first phases (namely, *sprouting* and *growing*) improved the implantation of the rice reducing the area damaged by the purple swamphen up to 37.8%. These first stages involve a crucial milestone for rice harvesting since a greater productive yield depends to a large extent on successful sowing and sprouting. Thus, only measures that reduce the movement of the purple swamphen between refuge zones and rice paddies are effective.

### Economic balance

Bearing in mind that administrative payments have reached 203,450 € per year and that the comparative reduction of damage at treatment plots with respect to the controls is 37.8%, the implementation of physical protection measures could save about 76,904 € annually to the government. From this figure, we subtract the costs of installation and maintenance of passive systems (vegetation management at the edges of the plots + physical barrier using plastic nets), which reached a unit price of 5.51 €/m, including the equipment used. If these preventive measures are applied throughout the perimeter of all wetlands with natural vegetation bordering rice crops, which is estimated at 23,070 m (considering the natural wetlands called *Canal Vell*, *Illa de Buda*, *Platjola-Migjorn*, *Riet Vell*, *Tancada* and *Encanyissada* where the purple swamphens are causing damage) the protection cost would reach 127,115.7 € during the first year. In subsequent years, the cost of clearing natural herbaceous vegetation and reinstalling the fence would have a unit cost of 1.2 €/m since the materials purchased during the first year are already available, which means an additional cost of 27,684 € per year. Therefore, the net savings in a five-years term should be calculated as the difference between the savings from compensatory payments during that period (5 years × 76,904 €) and the costs of implementation of measures (127,115.7 € for the first year and 27,684 € for the next four years). The final savings during five years of development of a protection program would be 146,668.3 €, which represents a total annual savings of 29,333.6 € for the management administrations.

### Legal implications

Harmonizing damage control and the protection of wild species is a paradigm in the fields of conservation biology and adaptive management (Conover, 2002; Redpath et al., 2013), due to the value of reconciling policies on socioeconomic development and biodiversity conservation (Treves, Wallace & White, 2009; Dickman, 2010). With the purpose of fulfilling this challenge, changes in the level of protection of the purple swamphen that could permit the lethal control of their populations, as requested by the agricultural stakeholders, were discarded a priori in accordance with existing regulations (Jefatura del Estado, 2007): the inclusion of wildlife species on lists of threatened or protected species is made by technical/scientific criteria, and not by matters of economic interest. A palliative solution to damage is the payment of economic compensations for subsidiary liability by the competent environmental administrations (Conover, 2002; Woodroffe, Thirgood & Rabinowitz, 2005). However, this is highly heterogeneous in terms of the judiciary interpretation of this responsibility, the way these compensations are taken into account by the different competent administrations and by how they are accepted or discouraged by the involved stakeholders (Nyhus et al., 2003).

### Management proposals

The inclusion of perimeter fencing and clearing of the peripheral vegetation of the rice fields within the scope of financing programs of agri-environmental measures from the Common Agricultural Policy (mainly FEADER and FEAGA) could be proposed as a management measure to reduce damage from purple swamphens. Moreover, the promotion and enhancement of agricultural insurances could contribute to mitigate wildlife damage in rice fields; to this end, publicly subsidized insurance lines are available in Spain to cover the economic costs associated with damage caused by the purple swamphen (Ministerio de Agricultura, Alimentación y Medio Ambiente (MAGRAMA), 2016).

Additionally, with the aim of understanding the impact more accurately, damage assessment and quantification systems should be improved for better compatibility and uniformity between plots, zones and even different wetlands. Technology could contribute to this goal, by means of an aerial photo series at a height lower to the ground than that used in our study. This could improve the quantification of the area of rice that had not properly matured due to the effects of purple swamphen damage. This new system, including well-regulated and accepted criteria by all involved stakeholders, would be more objective and viable than the current visual estimates made by rangers and technicians.

In addition to the tested measures and the frightening off of the birds, other actions that would reduce damage by the purple swamphen should be examined. New proposals could be aimed at decreasing bird densities, by means of live captures and translocations of purple swamphens to other wetlands, where the species is scarce or not present. This could be socially beneficial by demonstrating cooperation of the official bodies in the resolution of the conflict. Furthermore, it contributes to collaborations with population reinforcement or reintroduction programs that are being carried out in other countries and regions, following the procedure already performed with other common Iberian species such as the griffon vulture *Gyps fulvus* (Sarrazin, 2013). However, there are several aspects to this approach that would have to be examined. Legally, it would have to be ensured the adequate compliance of the purple swamphen protection status and, therefore, derogations have to be granted in compliance with EU environmental legislation (Article 9, Birds Directive 2009/147/EC). Moreover, the live removal of birds does not usually result in a definitive solution to the issue unless it is carried out continuously, which entails significant effort and the need to find favorable destination wetlands for the birds.

The role of public administrations in solving this conflict is essential: they have the means to implement vigilance and monitoring programs and can determine the actions to be taken, especially if the issue occurs in natural parks of great ecological value. This is already being exercised in the case of the Ebro Delta and should be strengthened to find sustainable solutions. In this sense, and as a previous positive example, the damage to rice fields by the greater flamingo *Phoenicopterus roseus* have been neutralized by the effort of ranger patrols, which scare the birds away in sensitive areas during the sprouting phases by means of sounds and lights.

## Supplemental Information

10.7717/peerj.4518/supp-1 Supplemental Information 1 Results of purple swamphen damage estimation. Results of purple swamphen *Porphyrio porphyrio* damage estimation of the different rice plots considered at the three study areas of the Ebro Delta (NE Spain). Several characteristics of each rice plot are included in the first five left columns such as the year of study, the total area of the plot, the length of borders of the plot in contact with reedbed *Phragmites* sp. and the type of plot distinguishing if preventive measures were put in place (Treatment) or not (Control). The percentage of area damaged in each plot during different stages of the rice development are also shown together with their subsequent log(10)+1 values.

10.7717/peerj.4518/supp-2 Supplemental Information 2 Results of damaged surfaces of studied plots.

10.7717/peerj.4518/supp-3 Supplemental Information 3 Table of results of home range of GPS-tracked purple swamphens.

## Additional Information and Declarations

### Data Availability

The following information was supplied regarding data availability: The two databases from which the statistical analyses were performed are provided as Supplemental Dataset Files.

## References

[ref-1] Battin J (2004). When good animals love bad habitats: ecological traps and the conservation of animal populations. Conservation Biology.

[ref-2] Bertolero A, Rivaes S, Mougeot F, Sánchez-Barbudo IS, Andree KB, Ibáñez C (2016). Sexing and ageing the Purple Swamphen *Porphyrio porphyrio* by plumage and biometry. Ardeola.

[ref-3] Blumstein DT (2006). Developing an evolutionary ecology of fear: how life history and natural history traits affect disturbance tolerance in birds. Animal Behaviour.

[ref-4] Boyd C, Punt AE, Weimerskirch H, Bertrand S (2014). Movement models provide insights into variation in the foraging effort of central place foragers. Ecological Modelling.

[ref-5] Canavelli SB, Branch LC, Cavallero P, González C, Zaccagnini ME (2014). Multi-level analysis of bird abundance and damage to crop fields. Agriculture, Ecosystems & Environment.

[ref-6] Conover M (2002). Resolving Human–Wildlife Conflicts: The Science of Wildlife Damage Management.

[ref-7] Dickman AJ (2010). Complexities of conflict: the importance of considering social factors for effectively resolving human–wildlife conflict. Animal Conservation.

[ref-8] Elphick CS (2000). Functional equivalency between rice fields and seminatural wetland habitats. Conservation Biology.

[ref-9] Elphick CS, Baicich P, Parsons KC, Fasola M, Mugica L (2010). The future for research on waterbirds in rice fields. Waterbirds.

[ref-10] Ernoul L, Mesléard F, Gaubert P, Béchet A (2014). Limits to agri-environmental schemes uptake to mitigate human–wildlife conflict: lessons learned from flamingos in the Camargue, Southern France. International Journal of Agricultural Sustainability.

[ref-11] Fasola M, Ruiz X (1996). The value of rice fields as substitutes for natural wetlands for waterbirds in the Mediterranean region. Colonial Waterbirds.

[ref-12] Food and Agriculture Organization (2012). FAO in emergencies: plant pests and diseases. http://www.fao.org/emergencies/emergency-types/plant-pests-and-diseases/en/.

[ref-13] Hemson G, Johnson P, South A, Kenward R, Ripley R, MacDonald D (2005). Are kernels the mustard? Data from global positioning system (GPS) collars suggests problems for kernel home-range analyses with least-squares cross-validation. Journal of Animal Ecology.

[ref-14] Ibáñez C, Curcó A, Riera X, Ripoll I, Sánchez C (2010). Influence on birds of rice field management practices during the growing season: a review and an experiment. Waterbirds.

[ref-15] Jefatura del Estado (2007). Ley 42/2007, de 13 de diciembre, del Patrimonio Natural y la Biodiversidad. Boletín Oficial del Estado.

[ref-16] Lane SJ, Azuma A, Higuchi H (1998). Wildfowl damage to agriculture in Japan. Agriculture, Ecosystems & Environment.

[ref-17] Louhaichi M, Borman MM, Johnson DE (2001). Spatially located platform and aerial photography for documentation of grazing impacts on wheat. Geocarto International.

[ref-18] Ministerio de Agricultura, Alimentación y Medio Ambiente (MAGRAMA) (2016). Orden AAA/1348/2016, de 29 de julio, por la que se definen los bienes y los rendimientos asegurables, las condiciones técnicas mínimas de cultivo, el ámbito de aplicación, los periodos de garantía, las fechas de suscripción y los precios unitarios en relación con el seguro de explotaciones de cultivos herbáceos extensivos, comprendido en el trigésimo séptimo Plan de Seguros Agrarios Combinados. Boletín Oficial del Estado.

[ref-38] Moorcroft PR, Lewis MA, Crabtree RL (1999). Home range analysis using a mechanistic home range model. Ecology.

[ref-19] Morgan JL, Gergel SE, Coops NC (2010). Aerial photography: a rapidly evolving tool for ecological management. BioScience.

[ref-20] Muthayya S, Sugimoto JD, Montgomery S, Maberly GF (2014). An overview of global rice production, supply, trade, and consumption. Annals of the New York Academy of Sciences.

[ref-21] Nyhus P, Fischer H, Madden F, Osofsky S (2003). Taking the bite out of wildlife damage the challenges of wildlife compensation schemes. Conservation in Practice.

[ref-22] O’Brien TG, Kinnaird MF (2008). A picture is worth a thousand words: the application of camera trapping to the study of birds. Bird Conservation International.

[ref-23] Pearlstine EV, Ortiz JS (2009). A Natural History of the Purple Swamphen (Porphyrio porphyrio).

[ref-24] Pellis SM (2011). Head and foot coordination in head scratching and food manipulation by purple swamp hens (*Porphyrio porphyrio*): rules for minimizing the computational costs of combining movements from multiple parts of the body. International Journal of Comparative Psychology.

[ref-25] Pernollet CA, Simpson D, Gauthier-Clerc M, Guillemain M (2015). Rice and duck, a good combination? Identifying the incentives and triggers for joint rice farming and wild duck conservation. Agriculture, Ecosystems & Environment.

[ref-26] Pierluissi S (2010). Breeding waterbirds in rice fields: a global review. Waterbirds.

[ref-27] Redpath SM, Young J, Evely A, Adams WM, Sutherland WJ, Whitehouse A, Amar A, Lambert RA, Linell JDC, Watt A, Gutiérrez RJ (2013). Understanding and managing conservation conflicts. Trends in Ecology & Evolution.

[ref-28] Sánchez-Guzmán JM, Morán R, Masero JA, Corbacho C, Costillo E, Villegas A, Santiago-Quesada F (2007). Identifying new buffer areas for conserving waterbirds in the Mediterranean basin: the importance of the rice fields in Extremadura, Spain. Biodiversity and Conservation.

[ref-29] Sánchez-Lafuente AM, Valera F, Godino A, Muela F (2001). Natural and human-mediated factors in the recovery and subsequent expansion of the Purple Swamphen *Porphyrio porphyrio* L. (Rallidae) in the Iberian Peninsula. Biodiversity and Conservation.

[ref-30] Sarrazin F, Papazoglou C, Charalambous C (2013). History of Griffon vulture reintroductions in France. Proceedings of the Griffon Vulture Conference.

[ref-31] Taylor B, del Hoyo J, Elliott A, Sargatal J, Christie DA, de Juana E (2016). Purple Swamphen (*Porphyrio porphyrio*). Handbook of the Birds of the World Alive.

[ref-32] Taylor B, Van Perlo B (1998). Rails: A guide to the Rails. Crakes, Gallinules and Coots of the World.

[ref-33] Toral GM, Figuerola J (2010). Unraveling the importance of rice fields for waterbird populations in Europe. Biodiversity and Conservation.

[ref-34] Tourenq C, Sadoul N, Beck N, Mesléard F, Martin JL (2003). Effects of cropping practices on the use of rice fields by waterbirds in the Camargue, France. Agriculture, Ecosystems & Environment.

[ref-35] Treves A, Wallace RB, White S (2009). Participatory planning of interventions to mitigate human–wildlife conflicts. Conservation Biology.

[ref-36] Van Huis A, Peshin R, Dhawan AK (2009). Challenges of integrated pest management in sub-Saharan Africa. Integrated Pest Management: Dissemination and Impact.

[ref-37] Woodroffe R, Thirgood S, Rabinowitz A (2005). People and Wildlife, Conflict or Co-Existence?.

