# Supplementary material for: Reconciling the conservation of the purple swamphen (Porphyrio porphyrio) and its damage in Mediterranean rice fields through sustainable non-lethal techniques
Source: PeerJ. 2018 Apr 24;6:e4518. doi: 10.7717/peerj.4518 (PMC5922229; doi:10.7717/peerj.4518)
Supplement: Supplemental Information 1 — Results of purple swamphen Porphyrio porphyrio damage estimation of the different rice plots considered at the three study areas of the Ebro Delta (NE Spain). Several characteristics of each rice plot are included in the first five left columns such as the year of study, the total area of the plot, the length of borders of the plot in contact with reedbed Phragmites sp. and the type of plot distinguishing if preventive measures were put in place (Treatment) or not (Control). The percentage of area damaged in each plot during different stages of the rice development are also shown together with their subsequent log(10)+1 values. [file peerj-06-4518-s001.docx]

Table S1. Results of purple swamphen *Porphyrio porphyrio* damage estimation of the different rice plots considered at the three study areas of the Ebro Delta (NE Spain). Several characteristics of each rice plot are included in the first five left columns such as the year of study, the total area of the plot, the length of borders of the plot in contact with reedbed *Phragmites* sp. and the type of plot distinguishing if preventive measures were put in place (Treatment) or not (Control). The percentage of area damaged in each plot during different stages of the rice development are also shown.

| Studied area | Year | Plot code | Total area of the plot (in ha) | Length in contact with reedbed of the plot (in m) | Type of plot | % area damaged during sprouting | % area damaged during growing | % area damaged during maturation |
| --- | --- | --- | --- | --- | --- | --- | --- | --- |
| Buda | 2013 | 1 | 2,67 | 100 | Treatment | 2,3 | 0,5 | 0,4 |
| Buda | 2013 | 2 | 2,56 | 112 | Treatment | 3,3 | 0,2 | 0,3 |
| Buda | 2013 | 3 | 3,14 | 75 | Treatment | 7,3 | 0,5 | 0,2 |
| Buda | 2013 | 4 | 2,70 | 95 | Treatment | 9,4 | 0,4 | 0,3 |
| Buda | 2013 | 5 | 3,07 | 131 | Control | 23,3 | 15,1 | 10,6 |
| Encanyissada | 2013 | 1 | 5,02 | 122 | Treatment | 7,2 | 2,3 | 2,4 |
| Encanyissada | 2013 | 2 | 5,56 | 143 | Treatment | 9,4 | 4,5 | 3,6 |
| Encanyissada | 2013 | 3 | 4,19 | 126 | Treatment | 5,3 | 0,7 | 0,7 |
| Encanyissada | 2013 | 4 | 5,19 | 153 | Treatment | 6,5 | 2,4 | 0,7 |
| Encanyissada | 2013 | 5 | 4,30 | 136 | Control | 4,9 | 6,2 | 4,2 |
| Platjola-Migjorn | 2013 | 1 | 2,31 | 250 | Treatment | 55,9 | 57,9 | 56,3 |
| Platjola-Migjorn | 2013 | 2 | 1,99 | 284 | Control | 45,0 | 58,4 | 73,9 |
| Platjola-Migjorn | 2013 | 3 | 1,52 | 295 | Treatment | 10,2 | 11,7 | 10,9 |
| Platjola-Migjorn | 2013 | 4 | 1,40 | 186 | Treatment | 12,0 | 3,9 | 5,2 |
| Platjola-Migjorn | 2013 | 5 | 0,68 | 197 | Treatment | 34,0 | 18,8 | 28,3 |
| Buda | 2014 | 1 | 3,00 | 131 | Control | 17,8 | 3,5 | 6,8 |
| Buda | 2014 | 3 | 2,70 | 95 | Treatment | 29,2 | 10,0 | 9,4 |
| Buda | 2014 | 5 | 2,80 | 75 | Control | 18,4 | 9,0 | 10,8 |
| Buda | 2014 | 7 | 2,50 | 112 | Treatment | 23,0 | 7,3 | 5,6 |
| Buda | 2014 | 9 | 2,60 | 100 | Control | 27,9 | 10,7 | 15,7 |
| Buda | 2014 | 11 | 2,50 | 56 | Treatment | 27,7 | 8,9 | 9,7 |
| Platjola-Migjorn | 2014 | 11 | 1,40 | 190 | Control | 17,0 | 4,5 | 5,5 |
| Platjola-Migjorn | 2014 | 9 | 1,55 | 300 | Treatment | 8,5 | 5,0 | 3,0 |
| Platjola-Migjorn | 2014 | 5 | 1,90 | 290 | Control | 47,0 | 19,8 | 22,7 |
| Platjola-Migjorn | 2014 | 13 | 3,92 | 280 | Treatment | 100,0 | 68,0 | 54,4 |
| Platjola-Migjorn | 2014 | 6 | 1,08 | 190 | Treatment | 37,4 | 3,5 | 3,2 |
| Platjola-Migjorn | 2014 | 12 | 0,69 | 186 | Control | 91,0 | 67,0 | 53,5 |
| Encanyissada | 2014 | 5 | 5,50 | 143 | Control |  |  | 6,4 |
| Encanyissada | 2014 | 7 | 4,80 | 122 | Treatment |  |  | 8,9 |
| Encanyissada | 2014 | 4 | 4,35 | 133 | Treatment |  |  | 11,2 |
| Encanyissada | 2014 | 2 | 5,50 | 159 | Control |  |  | 26,2 |
| Buda | 2015 | 1 | 2,55 | 94,5 | Treatment | 0,1 | 1,7 | 0,4 |
| Buda | 2015 | 2 | 3,12 | 91,0 | Treatment | 0,1 | 1,3 | 0,3 |
| Buda | 2015 | 3 | 3,05 | 92,2 | Treatment | 0,1 | 1,9 | 0,4 |
| Buda | 2015 | 4 | 3,05 | 76,0 | Treatment | 0,3 | 1,5 | 2,2 |
| Buda | 2015 | 5 | 2,68 | 113,2 | Treatment | 0,1 | 1,0 | 0,6 |
| Buda | 2015 | 6 | 2,59 | 109,6 | Control | 0,0 | 1,7 | 0,6 |
| Buda | 2015 | 7 | 2,64 | 109,1 | Control | 0,1 | 2,9 | 0,5 |
| Buda | 2015 | 8 | 2,76 | 101,6 | Control | 3,4 | 3,2 | 0,1 |
| Buda | 2015 | 9 | 2,87 | 103,4 | Control | 1,3 | 1,4 | 0,6 |
| Buda | 2015 | 10 | 2,55 | 55,8 | Control | 0,5 | 0,3 | 0,5 |
| Platjola-Migjorn | 2015 | 1 | 2,05 | 339,8 | Treatment | 0,5 | 0,1 | 0,6 |
| Platjola-Migjorn | 2015 | 2 | 3,00 | 0,0 | Treatment | 0,3 | 0,1 | 0,1 |
| Platjola-Migjorn | 2015 | 3 | 3,00 | 0,0 | Treatment | 1,2 | 0,1 | 0,2 |
| Platjola-Migjorn | 2015 | 4 | 2,99 | 0,0 | Treatment | 0,8 | 0,0 | 0,1 |
| Platjola-Migjorn | 2015 | 5 | 1,50 | 196,5 | Control | 1,5 | 0,7 | 1,1 |
| Platjola-Migjorn | 2015 | 6 | 1,43 | 195,5 | Control | 1,2 | 1,4 | 1,5 |
| Platjola-Migjorn | 2015 | 7 | 1,50 | 206,1 | Control | 1,9 | 1,2 | 2,1 |
| Platjola-Migjorn | 2015 | 8 | 1,43 | 199,6 | Control | 9,9 | 4,0 | 1,1 |
